# External auditory canal cholesteatoma: staging and treatment strategies

**DOI:** 10.3389/fneur.2024.1505108

**Published:** 2024-12-19

**Authors:** Guanwen He, Chang Lin, Zhongshou Zhu, Liangfeng Liu, Rifu Wei, Yiyun Hong

**Affiliations:** ^1^Ningde Clinical Medical College, Fujian Medical University, Ningde, China; ^2^Department of Otolaryngology, Ningde Municipal Hospital of Ningde Normal University, Ningde, China; ^3^Department of Otolaryngology Head and Neck Surgery, The First Affiliated Hospital of Fujian Medical University, Fuzhou, China

**Keywords:** external auditory canal, cholesteatoma, stage, classification, surgical procedures

## Abstract

**Objective:**

To investigate clinical staging systems and appropriate treatment strategies for external auditory canal cholesteatoma (EACC).

**Methods:**

We performed comparative analysis of the features of several staging schemes (Holt, Naim, Shin, Chang, Kaneda, Hn, and He) of EACC; retrospective analysis of the clinical data of 44 patients with primary EACC, and analyzed the prognosis.

**Results:**

He’s staging system (2019) was found to be particularly clear and practical. It defines each lesion stage comprehensively, reflecting the disease’s progressive nature. According to He’s staging, 2 stage I lesions underwent transcanal cholesteatoma removal (TCR). For 28 stage II lesions, TCR was performed, with 12 cases additionally undergoing canalplasty. Among the 10 stage IIIA lesions, 2 were managed through outpatient debridement, while the remaining 8 underwent TCR combined with partial mastoidectomy and canalplasty (including 2 cases with reconstruction). Three stage IIIC lesions underwent canalplasty and tympanoplasty following partial mastoidectomy. In one case of stage IV lesion, treatment involved mastoidectomy, canalplasty, and abscessectomy. Recurrence occurred in three patients with stage II lesions treated with TCR alone, while the remainder showed no recurrence. One stage IIIA lesion who underwent outpatient debridement only was unable achieve a completely dry ear, and another stage IIIC lesion whose perforated tympanic membrane did not heal due to a fungal infection.

**Conclusion:**

Clinicians can refer to He’s staging for the clinical staging of EACC to devise appropriate treatment strategies; minimally invasive surgical procedures can be flexibly chosen depending on the extent of lesion involvement, under the premise of complete resection of the lesion, but regular follow-up is crucial.

## 1 Background

Cholesteatomas are benign but expansive aggregations of keratinized squamous debris, which can damage surrounding spaces and eventually reach intracranial space, and usually occurs in the ear and brain (1). Cholesteatoma of the ear is mostly seen in the middle ear, while cholesteatoma of the external auditory canal (EAC) is considered to be rare or uncommon, and accounts for approximately 1/60–30 of all middle ear cholesteatomas (2). Its annual incidence has been reported to be approximately 1.5–3 cases per million individuals in Europe (3–6), and 23.3 cases in Ningde, Fujian Province (7). There are various staging (grading or classification) schemes for EAC cholesteatoma (EACC) (7–13), but none has been widely accepted to date. At the same time, there is no uniform treatment mode for EACC, for the same degree of lesions, the choice of treatment modality greatly varies among doctors (13). The low incidence of EACC has largely limited the clinical summarization of its characteristics. Thus, it is necessary to conduct an in-depth discussion on the clinical staging of this disease and the recommended therapeutic surgical modalities.

## 2 Materials and methods

### 2.1 Case selection

The Hospital Information System (HIS) was screened for cases with a diagnosis of EACC. The inclusion criteria were as follows: treatment between January 2013 and April 2023, high-resolution CT scan of the temporal bone before treatment, and follow-up for at least 1 year after surgery. The exclusion criteria included the following: incomplete medical records, recurrent EACC, and involvement of the middle ear such that it is difficult to distinguish between EACC and middle ear cholesteatoma. In all, 44 patients were included, of which 38 were hospitalized patients with a postoperative pathological diagnosis, and the remaining 6 were treated only on an outpatient basis without pathological examination. This study was reviewed and approved by the Research Ethics Committee of the Ningde Municipal Hospital Affiliated of Ningde Normal University with the approval number: 20221004, dated October 13, 2022.

### 2.2 Assessment methodology

(1) Clinical information, including age, sex, side of lesion, symptoms, location of bone erosion, and hearing before and after treatment, was retrospectively analyzed for all patients. (2) Representative staging (grading, classification) schemes for EACC retrieved from the literature included seven major staging schemes, namely James J. Holt (1992), Ramin Naim (2005), Seung-Ho Shin (2010), Jiwon Chang (2012), Shoji Kaneda (2016), Udayabhanu Hn (2018), and GW He (2019) (Table 1); the temporal bone CT of all patients was reviewed through our hospital’s Picture Archiving and Communication System (PACS); the treatment records of all patients were reviewed to analyze the treatment modalities and intraoperative findings. Finally, all the affected ears were staged using these seven staging schemes in combination with the pre-treatment temporal bone CT and intraoperative findings and the results were recorded. (3) Follow-up data were collected for all patients, and the interval between the date of the last follow-up and the date of the first treatment was defined as the follow-up duration.

**Table 1:** Clinical staging of external auditory canal cholesteatoma.

Stage	Staging (classification, grading)	Staging features
Holt (1992)	I: Superficial, saucerized defect; small localized pit II: Localized ear canal pocket III: Extension into the mastoid	The earliest staging scheme; relatively general; lesions beyond the temporal bone are not listed
Naim (2005)	I: Hyperplasia and hyperemia of the auditory meatus epithelium II: Localized inflammation of the hyperproliferated epithelium and adjacent periostitis. No destruction of the bony ear canal III: Destruction of the bony ear canal with sequestrated bone IV: Spontaneous destruction of the adjacent anatomical structures	Combines pathological evidence and includes early lesions (6 cases in this group could not be staged without pathologic examination); useful for understanding the development of disease; the difference in intraoperative sampling may misclassify stage II and III lesions
Shin (2010)	I: Limited to the EAC II: Invasion of the tympanic membrane and middle ear in addition to the EAC III: Defect caused in the EAC and inclusion of air cells in the mastoid bone IV: Inclusion of lesions beyond the temporal bone	Simple and easy to use; beneficial in treatment planning; Stage II to III lesions cannot reflect the progression
Chang (2012)	I: With flattening of bony external canal II: With partial destruction of inferior bony canal III: With total destruction of inferior bony canal IV- With bony destruction into the middle ear and mastoid cavity	Stage II and III lesions are regularly staged only for bone destruction in the inferior wall of the EAC (3 cases in this group could not be graded because the bony destruction was not located in the inferior wall of the EAC)
Kaneda (2016)	0: Only surface lesion without bony lesion I: Only bony erosion II: Bony deficit localized in the external auditory canal III: Invasion into the tympanic cavity, mastoid or combined IV: The adjacent anatomical structure complications	Stage I and II lesions are difficult to define (5 cases in this group the author could not be staged)
Hn (2018)	I: Without bone erosion and middle ear extension II: With bone erosion, ± middle ear extension III: With bone erosion + extension to adjacent structures (a) Without complications; (b) with complications	Simple and easy to use; beneficial in treatment planning
He (2019)	I: Invasion without bony lesions II: Invasion confined within the EAC, and possible bone erosion manifested as a rough edge or localized defect of the bone III: Invasion beyond the EAC, involving the mastoid air cells or tympanic cavity, but confined within the temporal bone A: Backward invasion into the mastoid air cells B: Inward or upward invasion into the tympanic cavity C: Invasion into the tympanic cavity and mastoid air cells IV: Invasion beyond the temporal bone or complications caused by the involvement of structures adjacent to the temporal bone	Defines each stage of the lesion clearly; include all degrees of lesions; reflects the progressive character of the disease; simpler and more practical in clinical practice; beneficial in treatment planning

## 3 Results

### 3.1 Clinical findings

All 44 patients had monaural lesions, with a male-to-female ratio of 16:28; patient age ranged from 7 to 81 years (median 34.5 years); and there were 17 left-sided and 27 right-sided cases. With regard to clinical symptoms, hearing loss (blockage, 38/44), otalgia (33/44), and otorrhea (18/44) were the three main symptoms. Bony erosion involved the posterior wall of EAC in 41 ears, inferior wall in 37 ears, anterior wall in 34 ears, and superior wall in 21 ears, with no bony erosion in 2 ears, single-wall bony erosion in 5 ears, and multiple-wall bony erosion in 37 ears. Intraoperative tympanic membrane perforation was found in 6 cases. A total of 38 patients underwent pre- and postoperative pure tone audiometry exam (6 patients with outpatient debridement did not undergo this test), and the mean values of pre-operative air conduction (AC) and air-bone gaps (ABG) were 41.3 ± 12.7 and 24.3 ± 6.7 dB HL, respectively (Table 2).

**Table 2:** Clinical data of external auditory canal cholesteatoma.

Sex	*n*
Male/female	16/28
Age	Year
Range	7–81
Median	34.5
Side	*n*
Left/right	17/27
Location of bone erosion	*n*
Anterior	34
Posterior	41
Superior	21
Inferior	37
Presenting symptoms	*n*
Hearing loss/sense of blockage	38
Otalgia	33
Otorrhea	18
Tinnitus	8
Pruritus	1
Pure-tone audiogram (*n* = 38)	Mean ± SD (dB)
Air conduction (pre-OP)	41.3 ± 12.7
Air conduction (post-OP)	25.2 ± 11.8
ABG (pre-OP)	24.3 ± 6.7
ABG (post-OP)	8 ± 5.3
Follow-up	Months
Range	13–120
Median	52

### 3.2 Staging and treatment modalities

In all, 44 patients were individually staged using the abovementioned staging schemes. Overall, 6, 3, and 5 patients had difficulty in being accurately staged using the Naim, Chang, and Kaneda’s staging systems, respectively. All stage I lesions (2 cases) in He’s staging scheme were managed with transcanal cholesteatoma removal (TCR); 16 out of 28 stage II lesions were managed with TCR (4 of which were performed only on an outpatient basis), and the remaining 12 were managed with TCR + canalplasty; 2 of 10 patients with stage IIIA lesions refused to be admitted to the hospital and were debrided only on an outpatient basis, and the remaining 8 were managed with TCR + partial mastoidectomy + canalplasty, and 2 of these also underwent EAC reconstruction; 3 stage IIIC lesions also underwent canalplasty + tympanoplasty after lesion resection (partial mastoidectomy); and 1 patient with stage IV lesion underwent mastoidectomy + canalplasty + abscessectomy because of the presence of a posterior cervical abscess. Of all the patients, 6 had perforated tympanic membranes, of which 3 (stage IIIC) underwent tympanoplasty and the remaining 3 (stage II) were had small perforations and were therefore not repaired (Table 3).

**Table 3:** Treatment and prognosis of external auditory canal cholesteatoma.

Stage^a^	Ears	Recommended treatment	Treatment in this study	Prognosis
I	2	TCR	TCR	No recurrence
II	28	TCR ± C	TCR = 16 (outpatient treatment = 4) TCR + C = 12 Small tympanic membrane perforation = 3, unrepaired	Recurrence = 3 (within 1 year post-operation; no recurrence after outpatient debridement) Tympanic membrane perforation self-healed within 6 months post-operation
IIIA	10	(TCR + M) + (C ± R)	TCR = 2 (outpatient treatment) TCR + M + C = 6 TCR + M + C + R = 2	No recurrence Wet ear = 1 (one of two cases treated in an outpatient clinic)
IIIB	0	TCR + T ± C ± R	—	—
IIIC	3	(TCR + M) + (C + T ± R)	TCR + M + C + T = 3 Tympanic membrane perforation = 3, repaired	No recurrence Tympanic membrane perforation unhealed = 1 (fungal infection)
IV	1	Flexible	TCR + M + C + abscessectomy	No recurrence

a According to He’s staging scheme; TCR, transcanal cholesteatoma removal; T, tympanoplasty; C, canalplasty; M, mastoidectomy; R, reconstruction.

### 3.3 Follow-up and prognosis

**Figure 1:**
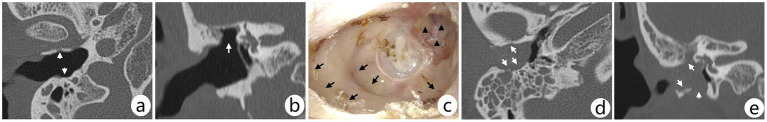
**(A–C)** CT scans and post-treatment otoscopic image of the right ear of a male patient. The patient refused hospitalization for surgery and chose to undergo TCR alone on an outpatient basis; three consecutive debridements over a 2-week period were followed by a CT scan of the temporal bone. Arrows in axial **(A)** CT show depressed areas, with a large bony defect in the anterior-superior bony wall of the EAC **(B)**; the defect (**C**, triangles) and enlarged or depressed areas (**C**, arrows) in the EAC can be further demonstrated on review of the otoscopy at 54 months post-treatment, with no recurrence in this case; however, the patient was unable achieve a completely dry ear. **(D,E)** Axial and coronal CT scans of a right ear lesion in a female patient. The arrows show that the lesion involves several bony walls of the EAC and a small number of air cells in the mastoid. Although the mastoid air cells were filled with shadows, it appeared more like infection than lesion invasion, which was intraoperatively confirmed; and the main body of the lesion was located in the EAC indicating that the lesion involves the middle ear rather than middle ear cholesteatoma involving the EAC.

All patients were followed up after treatment for 13–120 months (median 52 months). Three stage II patients who underwent TCR alone were found to have recurrence during the follow-up period, which occurred at postoperative 4, 8, and 10 months, respectively, and all of them underwent serial debridement on an outpatient basis. No recurrence was observed in the follow-up thereafter. Three stage II patients with small perforations of their tympanic membrane showed self-healing of the perforations within 6 months, whereas in the three other stage IIIC patients, one patient had a reperforation of the tympanic membrane because of a concomitant fungal infection after repair, and the remaining two had successful repairs. In addition, a stage IIIA patient refused to be hospitalized for surgery and chose to undergo TCR alone on an outpatient basis. This patient had a large bone defect in the anterior-superior portion of the EAC caused by the lesion (Figures 1A–C), which prevented him from achieving a complete dry ear. In all, 38 patients who pre-operatively underwent audiometric testing were reevaluated audiologically after the surgery (1–6 months) with mean AC and ABG of 25.2 ± 11.8 dB HL and 8 ± 5.3 dB HL, respectively.

## 4 Discussion

When EACC involves the middle ear, it needs to be differentiated from middle ear cholesteatoma involving the EAC (Figures 1D,E), which is more commonly observed in individuals with mastoid pneumatization insufficiency. In middle ear cholesteatoma involving the EAC, the main body of the lesion is located in the mastoid rather than the EAC and may lead to the destruction of bone in the posterior wall of the EAC, further leading to the involvement of the entire EAC. In contrast, in EACC, the involvement of multiple bony walls is common in early lesions; additionally, the disruption of the scutum caused by EACC occurs from the outside in rather than from the inside out. However, it is difficult to identify some extensive lesions.

The male-to-female ratio in this study was 1:1.75, the median age was 34.5 years, and the right-to-left side ratio was 1:1.6, which was similar to previous reports (7, 10, 13, 14). Among the clinical symptoms, complaints of hearing loss (blockage) were the most frequent, but acute otorrhea and otalgia were often the main reasons for patient visits to the clinic. Among the bony wall disruptions of the EAC, the posterior wall was the most involved followed by the inferior wall, which is in line with our previous case reports, and multiple bony wall involvement was also common (37/42). In most cases, the hearing loss caused by the lesion manifested as conductive deafness, and the hearing significantly improved postoperatively (*p* < 0.05), suggesting that the patients’ mechanical impairment of auditory conduction was alleviated after lesion removal.

There are several staging schemes for EACC (7–13). Holt’s (8) earliest proposed staging scheme was more general: when the lesion invades the tympanic cavity medially, destroying the auditory ossicle chain without invading the mastoid process, it is classified as a stage II lesion, whereas if the lesion progresses posteriorly and invades a small amount of the mastoid air cells, it can be classified as stage III, which may not be clinically recognized as a more serious lesion compared with the stage II lesions; furthermore, there is no separate classification for lesions that extend beyond the temporal bone. The greatest advantage of Naim’s et al. (9) staging scheme is that it combines pathological evidence of the disease and includes early lesions. Thus it is useful for understanding the development of EACC, but the difference in intraoperative sampling may misclassify stage II and III lesions (15), and six patients in this group received only outpatient debridement without pathological examination. Accordingly, these individuals could not be staged using this scheme. Shin’s et al. (10) staging is based primarily on the patient’s pre-treatment CT of the temporal bone, which is simple to use and clinically useful, and its stage II (involvement of the tympanic membrane and middle ear) and stage III (involvement of the mastoid air cells) lesions represent a different direction of focal invasion rather than a difference in the severity of the disease. Stage II and III lesions in Chang’s et al. (11) staging scheme were evaluated regularly only for bony destruction in the inferior wall of the EAC; in three of our cases, the bony destruction of the lesions was not located in the inferior wall of the EAC, and therefore, these individuals could not be staged using this scheme. In Kaneda’s et al. (12) staging scheme, it was difficult to differentiate between “bony erosion” and “bony deficit” in stage I and II lesions, and in the present study, there were five such patients in whom we had difficulty determining whether they were stage I or stage II lesions. Overall, the Hn et al. (13) staging scheme may be a good guide for the development of surgical strategies. A good staging scheme should have the following characteristics: it should incorporate all degrees of lesions; it should be based on basic routine investigations, such as CT, as far as possible; the individual stages should be clearly defined and it should be easy to differentiate between the various levels of lesions, and each stage should be able to reflect the progression or severity of the disease; and ideally it should be able to guide decision-making on treatment options or suggest a prognosis to a certain degree. The He’s et al. (7) staging scheme better meets the abovementioned characteristics and is worthy of reference and application by clinicians.

**Figure 2:**
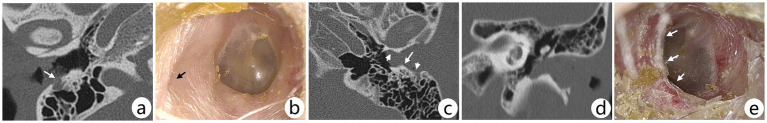
**(A,B)** Preoperative CT scan and postoperative otoscopic image of the right ear of a female patient. The arrow in axial CT **(A)** shows bone erosion in the posterior wall of the EAC; the patient underwent TCR; otoscopy **(B)** at postoperative 36 months showed a depressed area in the posterior wall of the EAC (arrow), but the overall transition of the EAC was gentle, and no lesion recurrence was observed. **(C–E)** Preoperative CT scan and postoperative otoscopic image of a stage II lesion in the left ear of a male patient. The short arrows in the axial **(C)** CT show depressed areas of the EAC, whereas the areas of bony plateau shown by the long arrows indicate an unsmooth transition in the posterior wall of the EAC; this patient underwent TCR only, and the bony plateau was not worn away, which can be seen on otoscopy **(E)** at postoperative 51 months. This may present a risk of recurrence, but this patient did not have a recurrence.

**Figure 3:**
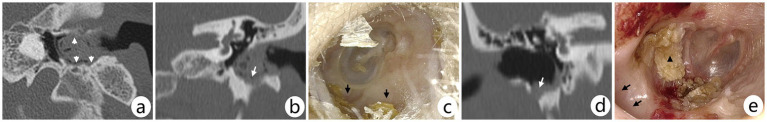
**(A–C)** Preoperative CT scans and postoperative otoscopic image of the left ear of a female patient. Arrows in the axial **(A)** and coronal **(B)** CT show lesions involving multiple bony walls of the EAC; the patient underwent partial mastoidectomy + canalplasty without EAC reconstruction; otoscopy **(C)** at postoperative 60 months shows depressed areas in the EAC (arrows), which may pose risk of recurrence, but there was no recurrence in this case. **(D,E)** Preoperative CT and postoperative otoscopic image of the right ear of a female patient. Arrow in coronal CT **(D)** shows bony erosion in the inferior wall of the EAC, creating a depressed area; otoscopy **(E)** at postoperative 8 months shows a depressed area (arrows) corresponding to the CT with recurrence of the lesion (triangle); the patient underwent TCR alone at first treatment, and debridement of the lesion was performed again on an outpatient basis when a recurrence is detected, suggesting that canalplasty should be performed at first treatment.

**Figure 4:**
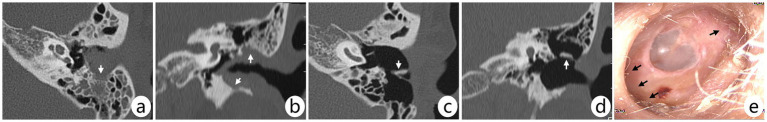
**(A–E)** Pre- and postoperative CT scans and postoperative otoscopic image of the left ear in a female patient. Arrows in preoperative axial **(A)** and coronal **(B)** CT show lesions involving all four bony walls of the EAC and mastoid, but the lesions did not invade the tympanic cavity (IIIA); the patient underwent mastoidectomy + canalplasty with reconstruction. Arrows in postoperative axial **(C)** and coronal **(D)** CT show reconstructed cartilage; otoscopy **(E)** at postoperative 96 months shows no recurrence of the lesion and significant enlargement of the deep EAC (arrow).

In our opinion, stage I lesions in He’s et al. (7) staging do not yet have bone destruction and can be treated with a single or consecutive multiple debridement. Stage II lesions have slight bone destruction but the lesion is still confined to the EAC, and during this time, if the transition of the EAC bone wall is gentle (Figures 2A,B), simple TCR is an appropriate choice of treatment (16, 17). If there is a large depression in the EAC, it is recommended to perform canalplasty to abrade the bone around the depression to form a gentle transition; otherwise, there is a risk of recurrence (16) (Figures 2C–E, 3A–C), and debridement can also become difficult after recurrence (Figures 3D,E). Stage III lesions have already exceeded the EAC but are still confined to the temporal bone. The lesions in stage IIIA mainly extend toward the mastoid, which can be resected like malignant tumors, i.e., under the premise of ensuring an appropriate “safety margin” (resection to healthy bone), as many air cells as possible should be resected; it is not necessary to pursue total resection of the mastoid air cells (Figure 4C), but it is necessary to ensure that the transition of the bony wall of the EAC is smooth and gentle. In our clinical observation, we have found that it is not required to strictly pursue the “large mouth and small bottom” EAC. In case of the involvement of a small number of air cells, if the transition of the EAC is smooth, the skin of the EAC can be reconstructed using only cartilage membrane, fascia, and skin fragments without reconstructing the bony wall; in case a large number of air cells are involved, if the wall of the EAC forms a depression or bony defect after resection of the lesion, the EAC wall can be reconstructed using bone fragments, cartilage, muscle, and other soft tissues (18–20) (Figure 4). The extent of lesion involvement should be determined by intraoperative viewing, and preoperative CT should be used as a reference, because sometimes the shadows in the mastoid air cells do not represent lesion involvement, but only infection (7). Stage IIIB lesions mainly extend toward the tympanic cavity, and the surgical approach here needs to be flexible depending on the extent of involvement. For example, the destruction of the tympanic membrane or even the auditory chain will require tympanoplasty to improve hearing. Stage IIIC lesions combine the tympanic cavity and mastoid air cells, and may require simultaneous partial or complete mastoidectomy + tympanoplasty + canalplasty (with or without reconstruction). Stage IV lesions extend beyond the temporal bone, and the choice of surgical procedure depends on the extent of involvement of the lesion.

It is worth noting that in areas of bone destruction, the bone in close contact with the cholesteatoma after resection can be smoothed with an electric drill to ensure “safe margins” in turn minimizing the recurrence risk of the lesion. In the case of EACC, the normal skin of the canal needs to be lifted up and properly protected. Although the patient’s wishes should be taken into consideration when choosing the treatment strategy, special care should be taken when adopting relatively conservative treatment strategies, such as TCR, because sometimes complete resection seen with the naked eye is not very reliable. Therefore, great importance should be given to the postoperative follow-up, and successive debridement should be performed when necessary to remove the residual or recurrent lesions in the EAC (16). For example, three cases in this group were found to have recurrence during follow-up within 1 year after surgery, all of which were treated with outpatient debridement and no recurrence was observed thereafter. Intraoperative perforation of the tympanic membrane may be seen in some patients, which may be caused by the direct destruction of the lesion or ischemia of the tympanic membrane caused by compression of the lesion. Intraoperatively, the diameter of the surgical suction tube tip can be used to estimate the size of the perforation. Our experience suggests that perforations less than 5 mm in diameter can be considered unnecessary to repair because small perforations tend to self-heal with the improvement of tympanic membrane blood supply after lesion resection and compression release. However, perforations larger than 5 mm can be repaired in stage I or II, which can be considered comprehensively in the light of the status of the perforated tympanic membrane, cholesteatoma lesion, and the condition of the patient (17).

In summary, the He’s staging scheme can be used to evaluate the EACC to formulate a treatment plan. After cleaning up the lesion, it is necessary to ensure that the transition of the bony EAC is smooth, and canalplasty can be considered for small depressions, whereas reconstruction of the EAC is required for larger defects. Lesions involving the mastoid air cells need partial or total mastoidectomy to remove the lesion up to healthy bone, and lesions involving the tympanic cavity need tympanoplasty. In clinical practice, treatment should be chosen flexibly according to the patient’s preference and the extent of lesion, and regular follow-up should be conducted.

## Data Availability

The original contributions presented in the study are included in the article/supplementary material, further inquiries can be directed to the corresponding authors.
